# Does clinical findings correlate with magnetic resonance imaging (MRI) findings in patients with temporomandibular joint (TMJ) pain? A cross sectional study

**DOI:** 10.4317/medoral.23501

**Published:** 2020-03-06

**Authors:** Cansu Gül Koca, Zeynep Gümrükçü, Elif Bilgir

**Affiliations:** 1 PhD, DDS, Assistant Professor. Department of Oral and Maxillofacial Surgery, Faculty of Dentistry, Uşak University, Turkey; 2 PhD, DDS, Assistant Professor. Department of Oral and Maxillofacial Surgery, Faculty of Dentistry, Recep Tayyip Erdoğan University, Turkey; 3 PhD, DDS, Assistant Professor. Department of Oral and Maxillofacial Radiology, Faculty of Dentistry, Eskişehir Osmangazi University, Turkey

## Abstract

**Background:**

Although magnetic resonance imaging (MRI) helps to clearly visualize the disorders in temporomandibular joint (TMJ), the relationship between cross-sectional and clinical findings has not been precisely established. The aim of this study was to evaluate the relationship between clinical symptoms and MRI findings in individuals with TMJ pain.

**Material and Methods:**

This cross-sectional study, conducted on the clinical and MRI findings of the patients, who applied to Uşak University, Oral and Maxillofacial Surgery Clinic with TMJ pain between the years 2016-2019. The primary predictor variables were MRI findings; disc position (normal, disc displacement with reduction (DDWR), disc displacement without reduction (DDWOR)), disc structural distortion (normal, folded, lengthened, round, biconvex, thick), condyle degeneration type (normal, moderate, severe) and joint effusion (JE) (absent, present). The primary outcome variable was pain, recorded on a visual analog scale (VAS) (numbered between 0-10). The other variables were demographic variables (age/gender). The relationship between clinical and MRI findings were statistically evaluated. The data were analysed by Kruskal Wallis and Mann Whitney U test. Chi-square (x2) test was used for categorical variable comparisons. *P* values < .05 were considered to indicate statistical significance.

**Results:**

Clinical and MRI records of 700 TMJ, from 350 patients with the mean age of the 31 (12-65) were evaluated in this study. Statistically significant differences were found between; disc position and pain, disc position and JE; JE and pain; disc structural distortion and pain; and disc structural distortion and disc position. JE was seen more common in DDWOR group. The most common disc distortion, seen in patients with JE, is the folded type.

**Conclusions:**

The present study can infer that pain is associated with disc position, JE, disc structural distortion, and DDWOR is associated with JE. Folded type disc is the most common disc type in TMJ with JE.

** Key words:**Internal derangement, TMJ, MRI evaluation, disc morphology, disc position, condyle degeneration, joint effusion.

## Introduction

Temporomandibular Joint (TMJ), which is one of the most complex joint in human body, has been defined as the location where the mandible articulates with temporal bone (1). Disorders occurring in this region are defined by American Academy of Orofacial Pain as a collective term of clinical problems consisting of Temporomandibular Joint Disorders (TMD), masticatory muscles, the associated structures or both (2,3).

Internal derangement (ID) (intra-articular irregularity) that can be seen even in asymptomatic individuals is the most common pathology in TMJ. The term derangement refers to a change in the normal course of movement of the TMJ, including the function of the articular disc. Since the structure and quality of TMJ do not change, this situation is different from degeneration (4).

The most common cause of internal derangement, also known as disc displacement, is the inadaptability between the condyle, the temporal bone and the articular disc (5). Disc displacement may be seen as the anterior reduction of disc with/without reduction (DDWR/DDWOR) and posterior displacement (PD) (6,7,8).

Degenerations of the condyle are known as osteophytes, erosion, sclerosis of the condyle, articular eminence or glenoid fossa (9).

MRI is a useful technique to detect internal derangement, as it allows the direct visualization of articular disc in both open and close mouth positions (10). Furthermore MRI allows detection of morphological and inflammatory changes in the TMJ. This technique is performed using radiofrequency (RF) waves that do not have ionizing properties. After the application of RF, the energy released from the body is determined and an MR image is generated on the computer (11).

Joint effusion, which is also known as the presence of inflammatory changes in retrodiscal tissue, can be detected by magnetic resonance imaging (MRI) evaluation with the presence of inflammatory changes in synovial membrane and retrodiscal tissue (12).

It has been reported that, the morphology and position of articular disc (AD) have an important role in the formations of the effusion (13). The morphology classification of AD is reported by Ottl *et al*. as; biconcave/normal, biplane/flat, thickened, biconvex, fragmented/destroyed (14).

All these disorders may develop in relation to each other in TMJ. Although there have been a few studies (7,9,12), which evaluated the correlation between a few MRI findings and clinical findings (between disc displacement and pain/joint effusion), the relationship between all MRI findings and clinical symptoms in TMJ still remains unclear.

The purpose of this study was to evaluate the correlation between clinical symptoms and MRI findings in patients with TMJ pain. The investigators hypothesize that there is a correlation between pain and articular disc position/disc distortion type, condyle degeneration type and joint effusion. The specific aim of the study was to evaluate the relationship between pain and the MRI parameters in TMJ.

## Material and Methods

- Method of sample selection

To address the research purpose, the investigators designed and implemented a cross sectional study with MRI evaluation. The study population was composed of successive patients records who applied for evaluation and management of TMJ pain between 2016 January and 2019 January. To be included in the study sample, patients had to be older than 12 year old and to have TMJ pain. Patients with a history of the presence of benign/malignant cyst/nodula, trauma or surgery in TMJ were excluded from the study.

Archived MRI images of the patients were evaluated by a single oral maxillofacial surgeon and confirmed by an oral maxillofacial radiologist over the records of patients. In case of contradiction, a 3-way evaluation was performed in consultation with another oral maxillofacial surgeon and agreement was reached.

- Study variables

Primary predictor variables were determined on MRI findings and grouped as; disc position, disc structural distortion types (disc morphology), condyle degeneration type and Joint effusion.

1.Disc position was classified under subheadings such as (7,15): 

Normal: Disc position was deemed as normal if the posterior band in relation to condyle was located between 11 and 12 o´clock.

DDWR: The disc is anterior to the condyle in the closed mouth position and returns to its normal position when jaw is opened.

DDWOR: The disc is anterior to the condyle in the closed mouth position and does not return to its normal position when the jaw is opened.

Posterior displacement: The posterior band of the disc is in apparent contact with the bilaminar zone and its anterior band is at a 2 o’clock or 3 o’clock position.

**Figure 1:**
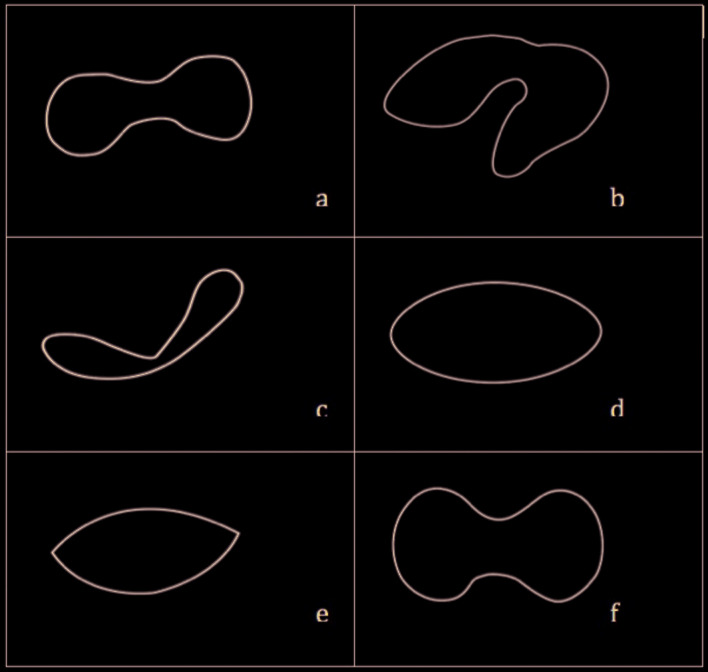
a) Normal: Disc is in biconcave form. b) Folded: Disc is in twisted form. c) Lengthened: Disc is in a longer form according to the normal form. d) Round: Disc is in round form. e) Biconvex: Disc is in a shuttle form. f) Thick: Disc is in a thicker form than normal form.

2. Disc structural distortion types (disc morphology): To assess the articular disc morphology, disc were categorised according it’s shape as follows; normal, folded, lengthened, round, biconvex, thick (Fig. 1) (16).

According to the classification of Murakami *et al*. (17) and a similar study (16) reported in the literature, disc shapes were categorized:

Normal: Murakami *et al*. (17) has been reported that the normal shape of the articular disc is in biconcave form (Fig. 1).

Folded: The twisted-looking disc was included in this group (Fig. 1).

Lengthened: Disc form, which is longer than normal length was included in this group (Fig. 1).

Round: The round disc form, which lost its concave structure was included in this group (Fig. 1).

Biconvex: The shuttle-like disc, which had lost its concave shape, was included in this group (Fig. 1).

Thick: Disc types thickened according to their normal size were included in this group (Fig. 1).

3. Condyle degeneration type: The degeneration of condyle was identified in three classes such as; normally, moderate degeneration (osteophtes, flattening, subchondral sclerosis, atrophy) or severe degeneration (erosion) (2).

4. Joint Effusion: Articular effusion has been identified as the presence of inflammatory changes in synovial membrane or retrodiscal tissue, and detected by a severe increase in the signal of T2 in MRI evaluation. In the present study, the categories were recorded as present or absent (12).

Primary outcome variable was pain, recorded on visual analog scale (VAS), in this study. The scores on the forms in which the right and left pain values of the patients were recorded using a callibrated scale between 0 and 10 were used (12).

The other variable category was demographic variables (age/gender) were obtained from recorded data.

- Data collection methods

All MRI images were obtained by a 1.5 Tesla Symphony Quantum or Avanto MRI (Siemens Medical, Erlangen, Germany) with a bilateral 80 mm diameter TMJ coil and 3 mm thick sections with a 190 mm field of view and 256 matrix. With the help of localizers; sagittal and coronal oblique plans were obtained. The following sequences were performed:

T1-weigheted images (200/11 TR/TE) to access disc position, disc morphology and osseous changes

T2- weighted images (1500/20 RTE/TE) to determine fluid and joint effusion

Both sequences were acquired in occlusion and maximum mouth opening. No motion artifacts were tolerated.

Reduction of the disc was evaluated by comparing T1 weighted MR images in open and closed mouth position and disc displacement was classified as disc displacement with/without reduction (15).

Articular disc morphology was classified as; normal, folded, lengthened, round, biconvex, thick post and perforation in the T1 sagittal plane (14,18).

- Data analysis

The SPSS 21.0 Package Data Program (SPSS 21.0 Software Package Program Inc., Chicago, IL) was used to evaluate the data. The data were analysed with the Kolmogorov-Smirnov test for conformity to normal distribution. The data were analysed by Kruskal Wallis H and Mann Whitney U test. Chi-square (x2) test was used for categorical variable comparisons. *P*<0.05 was considered statistically significant.

## Results

A total of 350 patient’s 700 TMJ data were included in the present cross sectional study with a mean age of 31 [12 – 65]; 179 male and 171 female (Table 1). Kruskal Wallis test was used for analysis of the parameters with more than 2 subgroups (disc position, condyle degeneration, disc structural distortion) and Mann Whitney U test was used for analysis of the parameters with 2 subgroups (JE). The results are summarized in the Table 1.

**Table 1:**
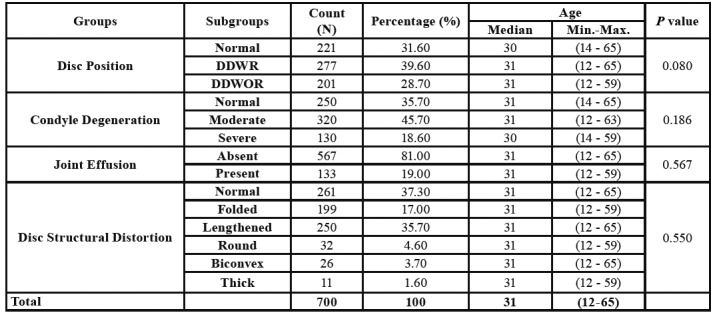
Distribution of the patients into the groups and relationship between age and disc position/condyle degeneration/joint effusion/disc structural distortion.

Any statistically significant difference was not found between age and other parameters (disc position, disc structural distortion, condyle degeneration and joint effusion) (*p*> 0.05).

Table 2 shows that, DDWR was found to be more common in males, while DDWOR was more common in females. This result was found to be statistically significant (*p*=0.02). Since the posterior disc displacement was seen only in one patient, PD was not included in the statistical analysis. With regard to the condyle degeneration classification, moderate type condyle degeneration was found to be more common in males and severe type found to be more common in females (*p*=0.003). Any statistically significant relationship was not found between disc structural distortion types and gender; and between joint effusion and gender (*p*=0.087, *p*=0.997).

**Table 2:**
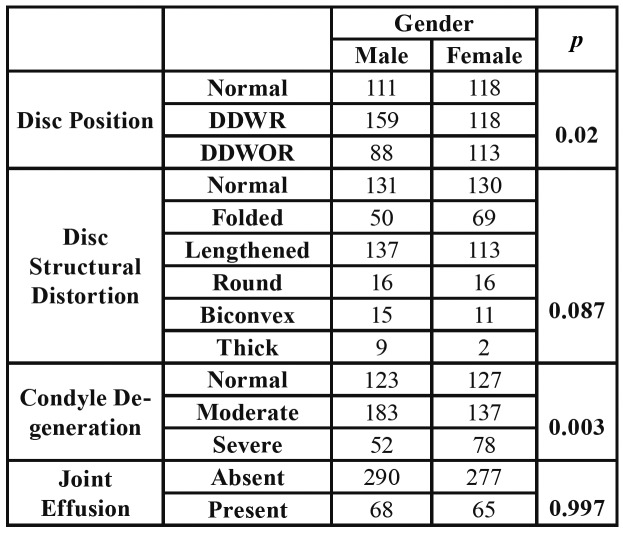
Relationship between gender and disc position/disc structural distortion /condyle degeneration/joint effusion.

There was no statistically significant difference found between pain and gender. (*p*=0.727).

Table 3 shows that patients with DDWR and DDWOR displayed higher pain levels than normal disc position group (*p*<0.001).

With regard to the condyle degeneration types, the highest pain value was found in the severe type and the lowest pain value was found in the normal type. Pain levels were significantly different between each group (Table 3).

**Table 3:**
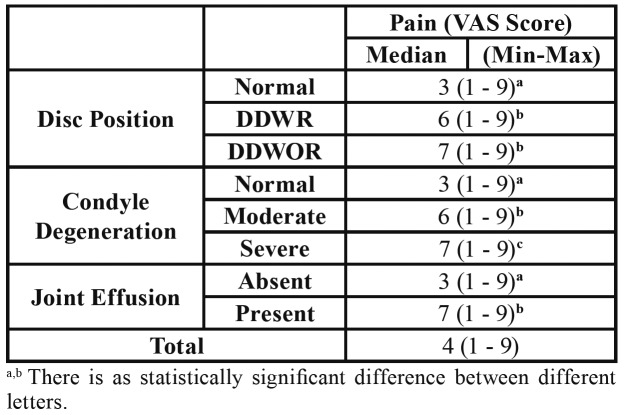
Relationship between disc position/condyle degeneration/joint effusion and pain.

With regard to the Joint effusion, the pain level in the joint effusion group was found to be significantly higher than the group without joint effusion *p*< 0.05 (Table 3).

Table 4 shows that, significantly higher pain values were found in folded type disc group according to the normal type disc group, in lengthened type disc group according to the normal type disc group and in the round type disc group according to the normal type disc group with regard to disc distortion types (*p*<0.001) (Table 4).

**Table 4:**
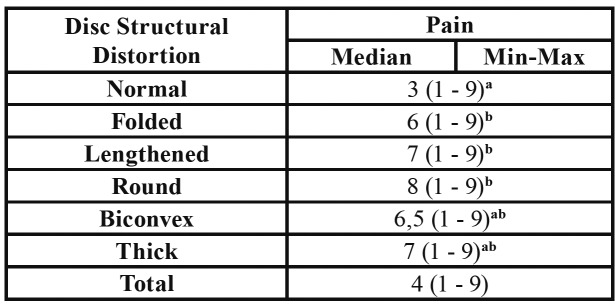
The relationship between pain and disc structural distortion.

Normal and round disc were found to be more common in the patients without JE according to patients with JE, and folded type disc found to be more common in patients with JE according to patients without JE (*p*<0.001). Since disc perforation was seen only in 1 patient, it was not included in the statistical analysis.

Normal type condyle degeneration was seen more common in normal disc position group, moderate type condyle degeneration was seen more common in DDWR group and severe type condyle degeneration was seen more common in DDWOR group. This difference, between each groups, was found statistically significant (*p*<0.05).

Normal disc form was seen more commonly in the normal disc position group, lengthened type disc was seen more commonly in DDWR group, and the folded type disc was seen more common in DDWOR group (*p*<0.05).

JE was seen more frequently in DDWOR group (53.20%) and the difference was statistically significant (*p*<0.05).

## Discussion

Temporomandibular joint disorders, known as a set of changing involving stomatognathic system especially in the masticatory musculature, TMJ or both. DDWR is one of the most frequent seen internal darangement type among the others, which may associated with/without pain. It has been known that, disc displacement may cause deformation in articular disc due to the mechanical overload, furthermore, this undesirable load causes release of inflammatory mediators in retrodiscal tissue and synovial membrane which is known as joint effusion and causes pain (12). Although, the relationship between pain and changes in MRI is an interesting subject, no certain conclusions have been achieved about this topic. To illuminate this deficiency in the literature, the present study was focused on evaluating the presence of pain in TMJ and evaluating the correlation of pain with MRI findings. The aim of the study was to investigate the relationship between clinical findings and MRI findings and thus to help to determine the treatment map as a result of this data analysis.

As a result of the study, we obtained results which support our study hypothesis: 1. Statistically higher pain values were found in patients with DDWR and DDWOR according to patients with non-deplased TMJ disc. 2. Pain levels were found higher in patients with folded, lengthened, and round type disc, than in patients with normal type disc. 3. It was found that the pain, felt in TMJ with high condyle degeneration, was significantly higher than the other degeneration groups 4. JE was found to be higher in patients with DDWOR. 5. JE was found to be significantly higher in TMJ with folded type joint disc.

A study evaluating the correlation between disc dislocation and pain was performed by Akdag *et al*. in 2018 (19). They evaluated 104 patients with unilateral TMJ pain. As a result of the study, any significant difference was not reported between DDWR and DDWOR group. As a result of the present study, higher pain scores were detected in DDWOR and DDWR than normal disc position group, and lowest pain values were detected for the normal disc position group. However, no significant difference was found between DDWR and DDWOR groups. The present study results are consistent with the study of Akdağ *et al*. in this regard.

It is known that the disc is located at the anterior of the condyle reversibly/irreversibly in the DDWR/DDWOR, and the extension and tension in the posterior ligaments are higher than the ligament of the disc with normal position. Anterior displacement of the disc results in overloading on the retrodiscal tissue, which, in turn, results in increased degeneration and inflammation of the joint due to the increase of free radical and nitric production in this region (20). Therefore, the higher pain values seen in DDWOR compared to DDWR due to this damage in posterior ligament may be attributed to this rationale and this is an explicable outcome for the current study.

There are several descriptions of JE in the literature. The definition of ‘Presence of fluid accumulation in TMJ’ is only one of them (12). It was reported that the diagnosis of JE was made by detecting bright T2 signals. However, literature knowledge still needs a clear definition of JE since it had various types from mild to severe. Larheim *et al*. (13). classified JE in four types as; normal, mild, moderate and marked. Contrary to other study reports (12,16), the JE was seen in a lower ratio of 19% in the present study. These lower JE rates, found in the present study, may be attributed to the evaluation of only the severe T2 signals and the lack of the evaluation of the lower/moderate signals.

Koh had reported (16) that there is no correlation between disc morphology and JE; in the present study, however, the results revealed a correlation between disc morphology and JE. The result is a new and valuable data for the literature, and the results may be attributed to the larger samples size, differences in materials/methods between Koh's, and the present study.

In a study by Murakami *et al*. (21), bilateral MRI effusion findings of patients with unilateral TMJ pain were evaluated. They reported no correlation between pain and effusion. In contrast with the results of the study of Murakami *et al*. (21) and *Pi*nto *et al*. (12), significantly higher VAS scores were detected in patients with JE according the patients without JE in the present study. The aforementioned studies were performed on 19 and 116 patient data, respectively. Considering that the current study was conducted on 350 patients with 700 TMJ data, the difference in the present study results is thought to may have been obtained owing to the number of the samples in the present study. The fact that it has been obtained from a large number of patients makes this study more sensitive and reliable among the other work.

Hosgör (22) evaluated the relationship between the pain and JE and reported that there was no statistically significant result with regard to pain in patients with moderate JE, while statistically significant results were reported for patients with marked JE. The present study results are consistent with the study results of Hosgör. It is possible to say that even if the study results are compatible, the acceptability level of the current study is stronger than the study of Hosgör, owing to be worked on a larger number of sample.

Takahara *et al*. (23) evaluated the relationship between MRI findings and pain and they were concluded that severe osteoarthritis, DDWOR and increase in joint fluid (JE) were associated with pain, but also they have reported that TMJ degeneration may be seen in asymptomatic individuals. In the present study, the finding of increase in pain with condylar degeneration is consistent with the study of Takahara *et al*. (23). The present study is one of the rare works in the literature to evaluate disc-condyle morphology, joint effusion, disc position, and pain parameters together in a single study. In this context, this study is a valuable study that will guide the evaluation and treatment of patients with TMJ pain by suggesting the relationship between clinical and MRI findings. The study was conducted retrospectively on available clinical and MRI data of the patients therefore, comparison of the MRI findings in painful and painless TMJ was not possible. Although this may be considered a limitation which weakens the study results, the present study is one of the most comprehensive studies focused on the relationship between the degree of clinical symptoms and MRI findings in the literature. Thanks to this, the current study results may shed light on clinicians in this regard.

## Conclusions

Statistically, this study infer that there is a strong correlation between JE and DDWOR. Folded type disc degeneration is the most common degeneration type seen in TMJ with joint effusion. Pain is correlated with DDWOR, severe condyle degeneration and JE. In the light of these findings, knowing that the degree of clinical symptoms correlates with internal darangement may provide information about the degree of TMJ disorder. Therefore, clinical symptoms may play a guiding and preventive role in the treatment of TMJ. Results of the study statistically proves the relationship between clinical data and MRI findings. However, more comprehensive, comparative and prospective clinical trials is planned for future studies that will eliminate the limitations of the current study.
